# Cellular modelling of Alström syndrome in human primary dermal fibroblasts and derived cells

**DOI:** 10.1186/2046-2530-1-S1-P102

**Published:** 2012-11-16

**Authors:** RK Semple, J-H Chen, RB Paisey, TG Barrett, M Hales

**Affiliations:** 1 University of Cambridge, UK; 2 Torbay Hospital, UK; 3 Birmingham University, UK; 4 Alström, UK

## 

Alström syndrome (AS) is a complex disorder whose manifestations include retinal degeneration, sensorineural hearing loss, cardiomyopathy, liver fibrosis, and severe insulin resistance. It is caused by biallelic loss-of-function mutations in the *ALMS1* gene, encoding a large centrosomal protein of poorly understood function. Although the syndrome encompasses several cardinal features of ciliopathies, primary cilia have been reported to be morphologically normal in primary cells from patients with AS. In order to dissect out the cellular pathology of AS in humans we have now, in a project led by Alström UK, assembled a bank of dermal fibroblasts from patients with AS. All 11 cell lines studied to date show normal primary cilia on serum starvation. 3/11 lines express near normal levels of ALMS1 protein at the centrosome despite biallelic *ALMS1* mutations, which will permit refinement of existing genotype-phenotype correlations in AS. We have also generated induced pluripotent stem cells that will be differentiated into cell types relevant to the organ-specific pathologies of AS including cardiomyocytes, hepatocytes and adipocytes. Finally we have used lentivirally-mediated expression of the adipose differentiation regulator PPARgamma2 to reprogramme human dermal fibroblasts to adipocytes. We have developed a highly efficient protocol to produce cells that accumulate triglyceride, show a pattern of gene expression consistent with adipocytes, secrete adiponectin and leptin, and respond physiologically to insulin. Collectively these developments constitute a valuable cellular resource for studying the cellular pathology of AS, and may form the basis of preclinical treatment screens in future.

